# Comparison of eutectic mixture of local anesthetics cream with dorsal penile nerve block using lignocaine for circumcision in infants

**DOI:** 10.12669/pjms.291.2944

**Published:** 2013

**Authors:** Sabeen Mujeeb, Jamshed Akhtar, Soofia Ahmed

**Affiliations:** 1 Sabeen Mujeeb, Dept. of Paediatric Surgery, National Institute of Child Health, Rafiquee Shaheed Road, Karachi-75510, Pakistan.; 2 Jamshed Akhtar, Dept. of Paediatric Surgery, National Institute of Child Health, Rafiquee Shaheed Road, Karachi-75510, Pakistan.`; 3 Soofia Ahmed, Dept. of Paediatric Surgery, National Institute of Child Health, Rafiquee Shaheed Road, Karachi-75510, Pakistan.

**Keywords:** Circumcision, EMLA, Anesthesia- circumcision, Penile nerve block, Pain, Infant

## Abstract

***Objective:*** Circumcision is a commonly performed surgical procedure but choice of anesthesia remained an issue of research and debate. This study was conducted to find out the effectiveness of the eutectic mixture of local anesthetic (EMLA) cream with dorsal penile nerve block (DPNB) using lignocaine, for reduction of pain during circumcision.

***Methodology:*** This was comparative study carried out in Surgical Unit B of National Institute of Child Health Karachi, from May 2008 to October 2008. Patients under six month of age were randomized in to two groups (EMLA and DPNB) of fifty patients each. The effectiveness of pain control was assessed by measuring the baseline heart rate (HR), respiratory rate (RR) and Neonatal infant Pain Scale (NIPS scale) before the start of procedure and measuring of these parameters for each step of circumcision. Independent sample t -test was used to compare means and repeated ANOVA was used to compare means of HR, RR, oxygen (O_2)_ saturations and NIPS.

***Results:*** The mean age in both the groups was 2.3 months. There was no statistically significant difference in baseline parameters in both the groups except the respiratory rate, which was significantly raised in DPNB group (33 breaths/min in EMLA and 38 in DPNB P < 0.04). During circumcision there was significant increase in heart rate in DPNB group, especially in step three and step four (p < 0.04). Oxygen saturation dropped in both the groups (baseline saturation 98% up to 91% in step 4). While assessing NIPS scores in both the groups, statistically significant difference was found between NIPS at step two and step four in two groups (p < 0.04).

***Conclusions:*** The overall pain control was equal in both the groups, although NIPS score was higher in DPNB in step two and four of circumcision. There was difference in application and cost. EMLA was easy to apply but has increased cost; while DPNB required expertise.

## Introduction

 Circumcision is one of the most commonly performed surgical procedures specially among Muslims and Jews.^1^ The understanding of the anatomy of the foreskin, pain perception and conditioning in infants changed the approach towards the procedure of circumcision in relation to anesthesia and analgesia.^2^ The neural pathways related to painful stimuli including the cortical and subcortical centers at which level pain is perceived, are well functional even at birth. Neonatal circumcision produces both physiologic as well as behavioral changes. Thus the need of some kind of anesthesia / analgesia for the procedure cannot be underestimated.^3^^-^^5^

 The procedure of circumcision is mostly performed under dorsal penile nerve block. This is simple, safe and effective technique to reduce pain. DPNB is often associated with significant complications in case of intravascular injection.^6^ The injection itself is also painful. Some form of analgesia which is painless to use, effective and produces no side effects is thus searched for.^7^

 EMLA cream is a eutectic mixture of local anesthetic. It contains 5% lidocaine-prilocaine cream (2.5% lidocaine and 2.5% prilocaine, Emla, Astra, Canada). Its properties allow increased absorption of the active ingredients through the skin.^8^ There are few studies available on the effectiveness of EMLA cream for neonatal circumcision.^9^ This study was undertaken to compare the effectiveness EMLA cream in terms of pain relief with that of DPNB in infants undergoing circumcision.

## Methodology

 Based upon the premise that EMLA cream would be equally effective in alleviating pain, in comparison to penile dorsal nerve block a study was conducted in the Surgical Unit B, Department of Paediatric Surgery National Institute of Child Health Karachi from May 2008 to October 2008. The study included children up to six months of age. Fifty patients each were assigned to EMLA and DPNB groups. The infants with history of hemorrhagic disease, congenital anomalies, allergic reactions or sensitivity to local application etc were excluded. Consent was taken. For assigning into each group balloting was used on operation day. All circumcisions were performed by Plastibell (Hollister Inc.) device in fully equipped operation theatre.

 The procedure was divided into four standard steps for the purpose of comparison. It included 1: application of forceps and separation of adhesions between prepuce and glans penis, 2: dorsal preputial incision after crushing of skin with hemostat, 3: insertion of Plastibell and tightening of encircling thread and finally 4: excision of prepuce skin. On day of circumcision the baseline physiological parameters such as HR, RR, and oxygen saturation were measured. In EMLA group 1-2 gm of EMLA cream was applied over the glans and prepuce. An occlusive Opsite (Smith & Nephew, Inc USA) dressing was applied, one hour prior to the procedure. In DPNB group injection was given by infiltrating 1ml of 1% (plain) lignocaine at penile base.

 An assessor recorded the physiological parameters and pain by using NIPS score during each standard step of the procedure. According to this scale Infants having intense pain would score between 5 and 6, infants receiving a somewhat effective anesthetic would score between 3 and 4, and infants receiving very effective anesthetic would score between 0 and 2.

 At the conclusion of the procedure baby was shifted to recovery room and kept under observation for one hour. On discharge, all patients were prescribed paracetamol drops. Baby was re-evaluated after one week. Side effects if any were recorded.

 The data was analyzed by SPSS-10. Frequency and percentages were computed for categorical variables. Mean and standard deviations were calculated for quantitative variables. Independent sample t-test was used to compare means. Repeated measure ANOVA was used to compare mean of HR, RR, oxygen saturation, NIPS score and for differences within subjects and between subject effects. P value <0.05 was considered significant.

## Results

**Fig.1:**
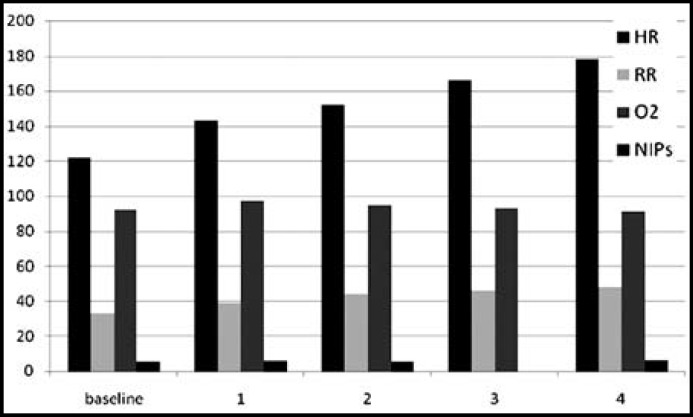
Comparison of Variables in EMLA Group

 Mean age of patients in each group was two months (range one month to six months). Mean baseline heart rate, respiratory rate, oxygen saturation and NIPS score observed are given in Fig.1. There was no statistically significant difference in base line parameters in both the groups except for the respiratory rate, which was significantly raised in DPNB group (<.01). There was no statistical difference in step one parameters. There was statistically significant difference in NIPS score between two groups which showed a higher NIPS score in DPNB group (p=.003) while rest of the parameters did not show any statistical significance in step 2.

**Fig.2:**
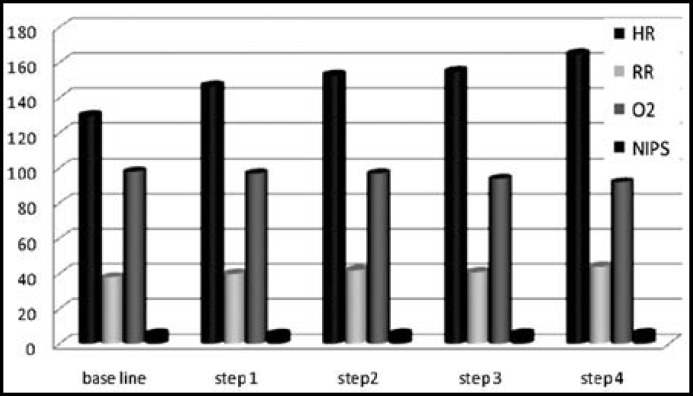
Comparison of Variables in DPNB Group.

 During step three, mean heart rate was 166/min in EMLA group and 155/min in DPNB group which was statistically significant ((p=0.04). NIPS score was 6 in EMLA group (range 5-7, SD=0.37) and in 6.14 in DPNB (range 4-7, SD=0.72). During step 4, heart rate in EMLA group was 178/min while it was 165/min in DPNB group which was statistically significant (p=.048). NIPS score was 6.06 in EMLA group (range 5-7) and 6.2 (range 5-7) in DPNB group which was statistically significant (p=.035). The data is given in Table-I. The parameters were also compared within the group at different steps and are shown in Fig. 1 and 2.

## Discussion

 According to WHO approximately 30% males are estimated to be circumcised globally.^1^ The safety of procedure is very much dependent upon pain management. The procedure is smooth in an adequately anesthetized baby. Thus a technique which is simple and least technically demanding is desirable. Though DPNB has been reported to be one such approaches it is still associated with complications though negligible like needle prick and minor incidence of local and systemic complications.^10^ EMLA cream thus appears to be more patient friendly by avoiding needle pricks.^11^

 The results of this study showed the mean baseline heart rate was slightly raised in DPNB group (eight beats) versus EMLA group which could be due to the pain experienced during the block. There was no significant difference in base line parameters in both the groups except the respiratory rate which was significantly raised in DPNB group (p <.01). Measurement of pain scores began three minutes after the DPNB. None of the reported studies measured the difference between the baseline physiological and behavioural parameters before and after the application of nerve block. It is observed that various factors can lead to physiological and behavioural changes in infants. Interpretation of pain score thus can be inaccurate.

 The mean HR during whole procedure in this study was159/min in EMLA group, for which the baseline was 122/min (difference of 37 beats per min) and 155/min in DPNB which increased from 130 beats per minutes (difference of 25 beats per min) at baseline level. Howard et al in their study compared heart rate and found that average rise in heart rate, above baseline was only 9 /min in DPNB group while it was >50/min in EMLA group.^12^ The heart rate increased five times greater in EMLA group than the DPNB group. In present study similar results with increase in heart rate up to 12 times higher in EMLA group noted. Benani et al compared EMLA with placebo and found attenuated pain response in infants receiving EMLA.^13^ In a study by Howard et al EMLA was compared with DPNB. Distress score was significantly higher in infants treated with the EMLA. They concluded that DPNB with lidocaine was a more efficacious means of providing anesthesia for neonatal circumcision.^12^

In index study it was observed that NIPS was not a good scoring system for the evaluation of pain in infants, although it is reported to be a non intrusive, replicable and objective tool and has shown high internal consistency in different studies.^14^ As evidenced by present study infants persistently had high NIPS scores as compared to study by Howard et al where it was 2.3 for DPNB and 4.8 for EMLA groups.^12^ This difference could be due the inter observer variability in assessing the behavioral component. The observation of present study was different from that reported by Howard et al in which they found that the maximum NIPS was observed in step one (adhenolysis), than foreskin cutting. Similar observation was made regarding respiratory rate and oxygen saturation. One interesting observation was the persistent maximum NIPS score throughout the procedure above the baseline level. Benini F et al, used different scale (neonatal facial coding scale), found that the lidocaine-prilocaine group spent less time crying during the procedure than the placebo group.^13^ Same are the observations and recommendations based upon present study.

 In 2004 Brady-Fryer B et al conducted a Cochrane systemic review to evaluate the efficacy of different analgesia during circumcision. They concluded that DPNB was the most frequently studied intervention and was the most effective for circumcision pain. Compared to placebo, EMLA was also effective, but was not as effective as DPNB. Both interventions according to them appear safe to be used for newborns circumcision. It was also noted that complete elimination of pain during circumcision was not achieved.^15^ Same were the findings in this study.

 An important learning through this study is related to steps of the procedure of circumcision. It appeared that the procedure of circumcision cannot be categorized into phases which could be assessed separately. Secondly NIPS score was difficult to apply in children already crying because of the separation from parents, environment and manipulation. It was difficult to differentiate whether behavioral changes were due to pain or simple crying. There was difficulty in recording both the physiological and behavioral components in this study.

 Although both EMLA cream and lidocaine appeared to have similar efficacy in pain control (though DPNB has probably better efficacy in pain control as suggested by the results) there exist a difference in their cost and method of application. EMLA cream costs more than an ampoule of lidocaine. EMLA cream also requires proper temperature requirement to be stored. For complete absorption and action, EMLA cream needs to be applied for at least one hour while lidocaine starts working in less than three minutes of application. The degree of absorption in EMLA cream cannot be predicted as it depends on many factors such as skin thickness and amount of ointment applied.^16^ There are less chances of systemic toxicity like methemoglobinemia in case of systemic absorption with EMLA cream.^17^ No measurable changes in the level of methemoglobin is reported in literature. The use of EMLA in premature and term infants is reported as safe.^18^ Similarly no untoward effects of EMLA cream were reported in this study.

**Table-I:** Comparative Data of Physiological Parameters (Mean values).

	*EMLA Group*				*DPNB Group*			
	*HR (n/min)*	*RR* *(n/min)*	*O*_2 _ *Saturation (%)*	*NIPS (n)*	*HR (n/min)*	*RR (n/min)*	*O*_2 _ *Saturation (%)*	*NIPS (n)*
Base Line	122	33	98	5	130	38	98	5.6
Step 1	143	39	97	5.6	147	40	97	5.8
Step 2	152	44	95	5*	153	42	97	6
Step 3	166*	46	93	6.06	155	41	94	6.1
Step 4	178*	48	91	6.06*	165	44	92	6.2

*Statistically significant

## Conclusions

 Both EMLA cream and DPNB were equally effective for pain control during circumcision however difference of cost and duration of application may be a constraint in using EMLA cream. 

## References

[1] (2007). Male circumcision: global trends and determinants of prevalence, safety and acceptability. WHO Library Cataloguing-in-Publication Data.

[2] Snell RS (1995). Clinical Anatomy for Medical Students.

[3] Halata Z, Munger B (1986). The neuroanatomical basis for the protopathic sensibility of the human glans penis. Brain Res.

[4] Light AR, Perl ER, Dyck PJ, Thomas PK (1993). Peripheral sensory systems. Peripheral Neuropathy.

[5] Rosen M (2010). Anesthesia for ritual circumcision in neonates. Paediatr Anaesth.

[6] Menif K, Khalidi A, Bouziri A, Hamdi A, Belhadi S, Jaballah NB (2011). Lidocaine toxicity secondary to local anesthesia administered in the community for elective circumcision. Fetal Pediatr Pathol.

[7] Shockley RA, Rickett K (J Fam Pract. 2011). Clinical inquiries. What’s the best way to control circumcision pain in newborns?.

[8] Cassidy KL, Reid GJ, McGrath PJ, Smith DI, Brown TL, Finley GA (2001). A randomized double-blind, placebo-controlled trial of the EMLA patch for the reduction of pain associated with intramuscular injection in four to six-year-old children. Acta Paediatr.

[9] Taddio A, Stevens B, Craig K, Rastogi P, David SB, Shennan A (1997). Efficacy and safety of lidocaine prilocaine cream for pain during circumcision. N Engl J Med.

[10] Soh CR, Ng SB, Lim SL (2003). Dorsal penile nerve block. Paediatr Anaesth.

[11] Taddio A, Ohisson K, Ohisson A (2000). Lidocaine-prilocaine cream for analgesia during circumcision in newborn boys. Cochrane Database Syst Rev.

[12] Howard CR, Howard FM, Fortune K, Generelli P, Zolnoun D, tenHoopen C (1999). A randomized, controlled trial of a eutectic mixture of local anesthetic cream (lidocaine and prilocaine) versus penile nerve block for pain relief during circumcision. Am J Obstet Gynecol.

[13] Benini F, Johnston C, Faucher D, Aranda J (1993). Topical anesthesia during circumcision on newborn infants. J Am Med Assoc.

[14] Lawrence J, Alcock D, McGrath P, Kay J, MacMurray S, Dulberg C (1993). The development of a tool to assess neonatal pain. Neonatal Network.

[15] Brady–Frayer B, Weibi N (2004). Pain relief for neonatal circumcision. Cochrane Database Syst Rev.

[16] Gazarian M, Taddio A, Kent G, Koren G (1995). Penile absorption of EMLA cream in piglets: implications for use of EMLA in neonatal circumcision. Biol Neonate.

[17] Couper RT (2000). Methaemoglobinaemia secondary to topical lignocaine/ prilocaine in a circumcised neonate. J Pediatr Child Health.

[18] Biran V, Gourrier E, Cimerman P, Walter-Nicole E, Mitanchez D, Carbajal R (2011). Analgesic effects of EMLA cream and oral sucrose during venipuncture in preterm infants. Pediatrics.

